# Photocatalytic Degradation of Tetracycline Hydrochloride via a CdS-TiO_2_ Heterostructure Composite under Visible Light Irradiation

**DOI:** 10.3390/nano8060415

**Published:** 2018-06-08

**Authors:** Wei Li, Hao Ding, Hua Ji, Wenbin Dai, Jianping Guo, Gaoxiang Du

**Affiliations:** 1 Beijing Key Laboratory of Materials Utilization of Nonmetallic Minerals and Solid Wastes, National Laboratory of Mineral Materials, School of Materials Science and Technology, China University of Geosciences (Beijing), 100083 Beijing, China; ptliwei@163.com (W.L.); dugaoxiang@cugb.edu.cn (G.D.); 2 State Key Laboratory of Solid Waste Reuse for Building Materials, Beijing Building Materials Academy of Sciences Research, 100041 Beijing, China; daiwenbin5210@163.com (W.D.); guojianping@bmtbj.cn (J.G.); 3 Suez Water Treatment Company Limited, 100026 Beijing, China; huajkl833@163.com

**Keywords:** CdS-TiO_2_ heterostructure composite, photocatalyst, tetracycline hydrochloride

## Abstract

A photocatalytic active CdS-TiO_2_ heterostructure composite was prepared by hydrothermal method and its morphology and properties were characterized. Results indicate that the CdS nanoparticles deposited on the surface of a TiO_2_ nanoparticles, which was in anatase phase. The particle scale of both of the components reached approximately 15 nm. In comparison to pure TiO_2_ (410 nm), the light absorption edge of the heterostructure composite was 550 nm, which could extend the light response from UV to the visible region. Under visible light irradiation, the degradation efficiency of tetracycline hydrochloride by the CdS-TiO_2_ composite achieved 87.06%, significantly enhancing photocatalytic activity than the as-prepared pure TiO_2_ and commercial TiO_2_ (Degussa P25). This character is attributed to the synergetic effect of these two components in the absorption of visible light.

## 1. Introduction

As one kind of N-type semiconductor, titanium dioxide (TiO_2_) has been extensively studied for its excellent properties, such as its low cost, chemical stability, non-toxicity, and high photocatalytic activity [1,2,3,4,5]. Of these merits, photocatalytic application has attracted a great attention due to its potential for efficiently exploiting solar energy to solve the global energy crisis [6,7] and environmental pollution [8]. Nevertheless, it can only absorb ultraviolet (UV) light and cannot be excited by visible light irradiation for its wide band gap energy (≥3.2 eV) [9] and fast recombination of photogenerated electron-hole pairs [10,11]. To take full advantage of visible light, the light response of the semiconductor must be extended from UV to the visible region. To accomplish this, efforts have been made, such as metal doping [12,13], nonmetal doping [14,15,16,17], reducing its band gaps by hydrogenation [18,19] and sensitizing with a low band gap semiconductor material [20,21,22,23,24,25,26]. Among previous methods, cadmium sulfide (CdS) nanocrystal was widely used to sensitize TiO_2_ for its high activity and quantum efficiency in the visible light region as a result of its reasonable band-gap energy (about 2.3 eV) [27,28,29,30,31]. Another advantage for this combination of CdS and TiO_2_ is that the photogenerated electrons in the conduct band of CdS can be transferred to the conduct band of TiO_2_ while leaving holes in CdS, which effectively prolong the lifetime of the photogenerated charge carriers [32,33,34].

There are numerous reports about CdS-sensitized TiO_2_ of binary semiconductor composites, most of which are about bulk-TiO_2_ and micro-CdS as well as how CdS plays a role in quantum dots [27,32,35,36]. Although photogenerated electrons could be excited from valence band to conduction band of CdS, it is difficult for these electrons to transfer to the conduction band of TiO_2_ as a result of the limited contact area between CdS and TiO_2_, leading to low photocatalytic efficiency. Until now, little attention has been paid to the attempt of two components in a nano-scale particle. Herein, we present a preparation of binary semiconductor composites, using Titanium oxysulfate as titanium precursor. In this composite, CdS nanoparticles are uniformly decorated on the surface of TiO_2_ and both particles are similar in size. CdS and TiO_2_ contacted closely in nano-scale rather than aggregating respectively. This nanostructure provides a higher degree of contact area between CdS and TiO_2_ than traditional binary CdS-TiO_2_ nanostructures and demonstrates high photocatalytic activity in degradation within a tetracycline hydrochloride solution. Our research provides an insight in designing highly efficient visible-light photocatalysts which are based on a heterostructure as well as better understanding of the photocatalytic reaction mechanism.

## 2. Experimental Procedure

### 2.1. Raw Materials and Reagents

**Figure 1:**
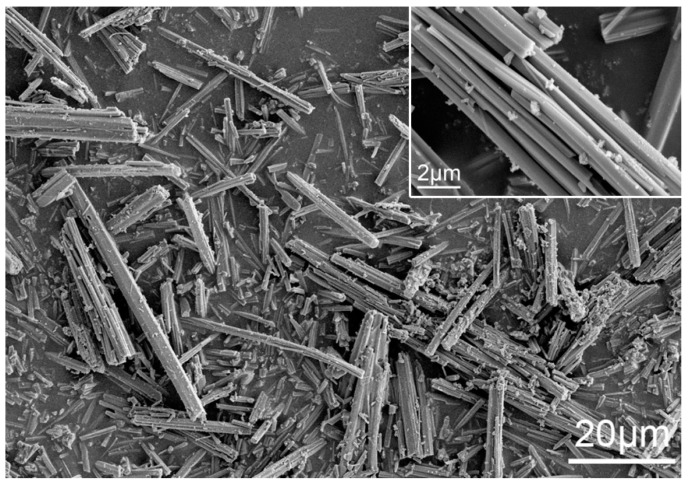
Micrographs of titanium precursor-Titanium oxysulfate.

In this study, thioglycolic acid was purchased from Tianjin Guangfu Fine Chemical Research Institute (Tianjin, China). Other reagents, including titanium precursor Titanium oxysulfate—sulfuric acid hydrate (TiOSO_4_·*x*H_2_SO_4_*·x*H_2_O), ethanol, cadmium acetate, and sodium sulfide, were purchased from Aladdin Chemical Co., Ltd. (Shanghai, China). All reagents were of analytical grade, without further purification. Figure 1 shows scanning electronic microscope (SEM) images of titanium precursor (TiOSO_4_·*x*H_2_SO_4_·*x*H_2_O).

### 2.2. Preparation Method

#### 2.2.1. Synthesis of TiO_2_ Nanoparticles

8.00 g of Titanyl sulfate (TiOSO_4_) was added to 145 mL absolute ethanol (molar ratio = 1:50) and kept magnetic stirring for 24 h. After mixing evenly, 40 mL suspension was extracted and added to a 50 mL autoclave. The solvothermal treatment in the autoclave was processed at 110 °C for 24 h. Afterwards, the white precipitate was filtered using a vacuum filter holder (Tianjin Jinteng Experimental Equipment Co., Ltd., Tianjin, China), washed thoroughly with absolute ethanol, dried in vacuum oven (Gongyi Yuhua Instrument Co., Ltd., Gongyi, China) at 100 °C, and finally calcined in a furnace (Beijing Zhongke Aobo Technology Co., Ltd., Beijing, China) at 550 °C for 3 h [37].

#### 2.2.2. Preparation of CdS-TiO_2_ Heterostructure Composites

To load the CdS onto the TiO_2_ surface, a reported procedure in the literature was introduced [38]. 0.16 g of TiO_2_ nanoparticles was put into 50 mL deionized water and stirred for 0.5 h. Next, 4 mL of Cd(CH_3_COO)_2_ solution (0.1 M), 300 μL of analytical grade thioglycolic acid and 4 mL of Na_2_S solution (0.1 M) were added into the suspension, respectively. After magnetic stirring for 1 h, 40 mL of the suspension was added to a 50 mL Teflon-lined stainless steel autoclave (Shanghai Kesheng Instrument Co., Ltd., Shanghai, China) and heated at 160 °C in an oven (Gongyi Yuhua Instrument Co., Ltd., Gongyi, China) for 14 h. The autoclave was then cooled at room temperature. Afterwards, the product was centrifuged (Shanghai Anting Scientific Instrument Factory, Shanghai, Chian) and then washed with deionized water. Subsequently, the yellow powder was dried at 60 °C in a vacuum oven for 10 h. For comparison, a pure CdS nanoparticles was also synthesized following the same protocol described above, without the addition of TiO_2_.

### 2.3. Characterization

#### 2.3.1. Characterization of Structure and Morphology

The products were characterized by X-ray diffraction (XRD) in reflection mode (Cu Kα radiation) on an UltimaIV X-ray diffractometer (Rigaku, Japan) at a scanning rate of 4°/min in 2θ ranging from 15° to 85° (λ = 0.15418 nm).

The particle size and morphology was visualized using a field-emission scanning electronic microscope (FESEM) (Gemini SEM 500, Carl Zeiss, Jena, Germany) with the energy dispersive X-ray (EDX) (X-Max Extreme, Oxford Instruments, Oxford, UK) spectrum analysis capability, operating at accelerating voltages of 20 kV.

Transmission electron microscopy and high-resolution transmission electron microscopy (HRTEM) (FEI Tecnai G^2^ F30 TEM, Hillsboro, OR, USA) The electron accelerating voltage was 300 kV. A small amount of the sample was first dispersed in alcohol by sonication. One drop of the suspension was then dropped onto a thin, hole-filled carbon film. The girds were then dried under an infrared lamp (Shanghai Kesheng Instrument Co., Ltd., Shanghai, China) for 10 min before TEM measurement.

The optical properties of CdS-TiO_2_ heterostructure composite as well as pure TiO_2_ and pure CdS nanoparticles were investigated using an Ultraviolet-visible Lambda 365 diffuse reflectance spectrophotometer (PerkinElmer, Waltham, Massachusetts, USA), which was equipped with a Labsphere diffuse reflectance accessory using a standard white board as a reference. In addition, the adsorption values of the tetracycline hydrochloride concentration were also measured by Lambda 365 UV-vis spectrometer.

#### 2.3.2. Photocatalytic Degradation of Tetracycline Hydrochloride under Visible Light Irradiation

The photocatalytic degradation of tetracycline hydrochloride was carried out at room temperature in an 80 mL self-designed quartz photochemical reactor containing 50 mL of aqueous solution (50 mg/L). 50 mg of sample was dispersed in the solution and then the suspension was stirred for 1 h to reach the adsorption-desorption equilibrium. All reactors were irradiated using a 500 W Xenon lamp (Beijing NBeT Technology Co., Ltd., Beijing, China) with an ultraviolet filter (λ > 400 nm) (Nbet) to cut off UV light [11,39]. 5 mL of the reacted solution was extracted from the quartz reactor at a given irradiation time interval and then measured using a UV-vis spectrometer at the maximal absorption wavelength of 356 nm to calculate the degradation efficiency (*C/C_0_*).

## 3. Results and Discussion

### 3.1. Structure and Morphology of CdS-TiO_2_

#### 3.1.1. Phase and Chemical Constitution of CdS-TiO_2_ Heterostructure Composite

**Figure 2:**
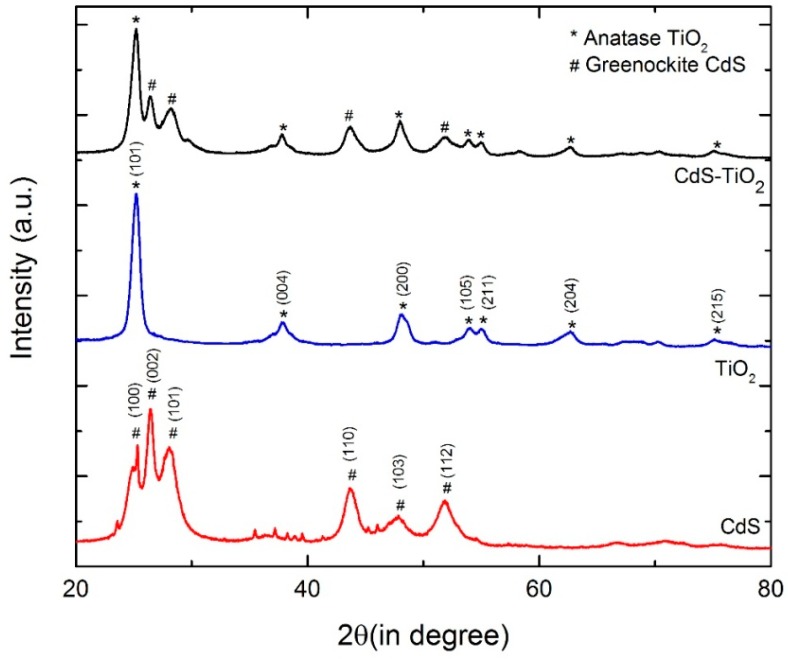
X-ray diffraction (XRD) patterns of CdS, TiO_2_, and CdS-TiO_2_ composite.

Figure 2 presents the diffraction patterns of the pure TiO_2_ nanoparticles, the pure CdS nanoparticles, and the CdS-TiO_2_ heterostructure composite with 50% (*w/w*) of CdS. For the TiO_2_ nanoparticles, the diffraction peaks at 25.3°, 37.80°, 48.0°, 53.9°, 55.1°, 62.7°, and 75.1° in the XRD pattern can be attributed to the (101), (004), (200), (105), (211), (204), and (215) crystal planes of anatase TiO_2_ (JCPDS no. 21-1272) [20], respectively. From the XRD patterns of CdS, it can be seen that the diffraction peaks at 2θ values of 24.8°, 26.5°, 28.2°, 43.7°, 47.9°, and 51.9° are in good agreement with the (100), (002), (101), (110), (103), and (112) crystal planes of the hexagonal structure of the CdS (JCPDS no. 75-1545), respectively [40]. All the XRD patterns of the CdS-TiO_2_ are consistent with both the anatase TiO_2_ and greenockite CdS, indicating that the heterostructure composite is composed of the two phases. We also calculated the average crystal sizes of greenockite CdS, anatase TiO_2_, and CdS-TiO_2_ composite nanoparticles via the peak width of the (002) plane of greenockite CdS and the (101) plane of anatase TiO_2_ by Scherrer’s formula (shown as follows). The results are shown in Table 1 [41].

$$D=\frac{K\lambda }{\beta \cos\theta }$$

where K is a constant (shape factor, about 0.89), *λ* is the X-ray wavelength, *β* is the FWHM of the diffraction line, and *θ* is the diffraction angle.

The results indicate that the average particle sizes of pure CdS and TiO_2_ are close and without any change after forming the composite, which was also able to meet the preparation requirements of CdS-TiO_2_ heterostructure.

#### 3.1.2. Microstructure of CdS-TiO_2_

**Figure 3:**
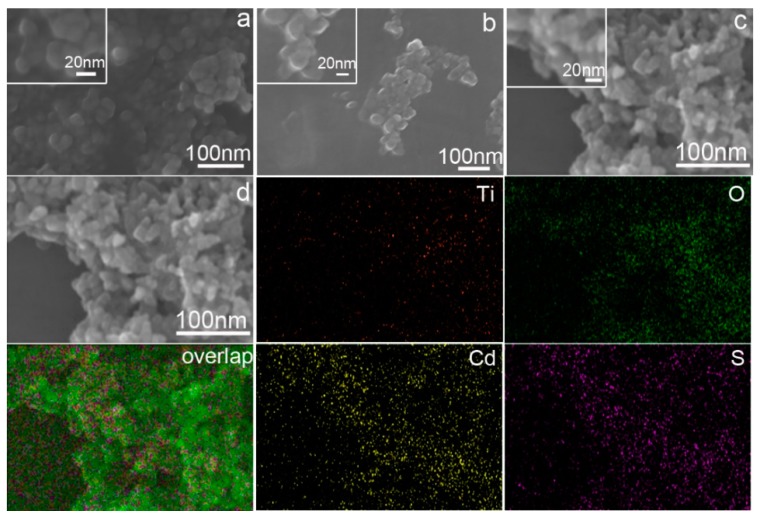
Scanning electronic microscope (SEM) micrographs of TiO_2_ (**a**); CdS (**b**); and CdS-TiO_2_ composite (**c**) and energy dispersive X-ray (EDX) mapping results of the composite (**d**).

The morphology of the pure TiO_2_ nanoparticles, CdS nanoparticles, and CdS-TiO_2_ composites have been analyzed by FESEM. Spherical morphology, as depicted in Figure 3a, shows uniformity with a diameter of 15 nm. As shown in Figure 3b, the CdS nanoparticles show an oval-liked morphology in the SEM image and their diameter are about 20 nm. Figure 3c shows that, although CdS were deposited, the two components are similar in size, making it difficult to distinguish them via SEM image. Figure 3d shows the distribution of the four elements (Ti, O, Cd, and S) in the composite. Distribution overlapping indicates a uniform combination of CdS and TiO_2_. The signals of Cd and S are greater than Ti and O, indicating that the TiO_2_ particles were decorated by CdS.

**Figure 4:**
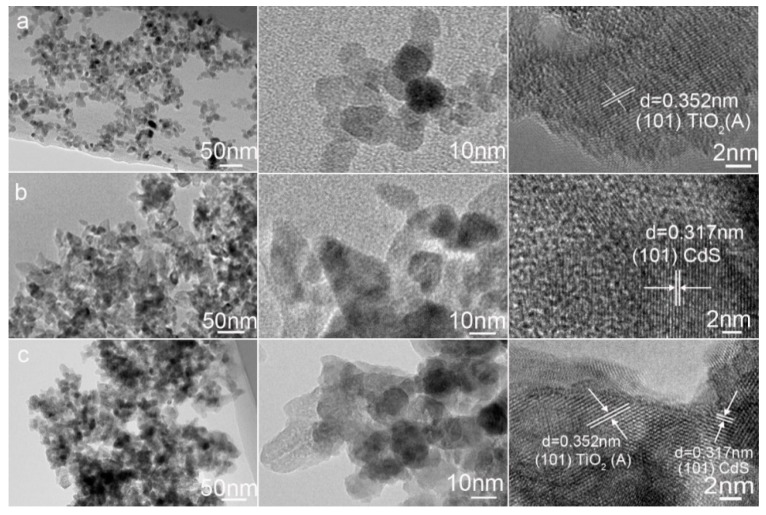
Transmission electron microscopy (TEM) micrographs of pure TiO_2_ (**a**); pure CdS (**b**) and CdS-TiO_2_ composite (**c**).

Transmission electron microscopy (TEM) was also applied to verify the fabrication of the CdS-TiO_2_ heterostructure. As seen in Figure 4a, the TiO_2_ nanoparticles are about 15nm in diameter, which agrees well with the SEM observations. In Figure 4b, the CdS nanoparticles are olive-like in shape with a diameter of about 20 nm. However, in Figure 4c, the CdS nanoparticles aggregates with TiO_2_. The representative high resolution TEM (HRTEM) images revealed that the lattice spacing of the CdS-TiO_2_ composite was 0.317 nm and 0.352 nm, which corresponded well to the (101) plane of greenockite CdS and the (101) plane of anatase TiO_2_, respectively. It further confirmed the interfacial junction between CdS and TiO_2_, in which CdS was closely attached to that of TiO_2_.

#### 3.1.3. Formation Mechanism of the TiO_2_ Nanoparticles and CdS-TiO_2_ Heterostructure Composite

**Figure 5:**
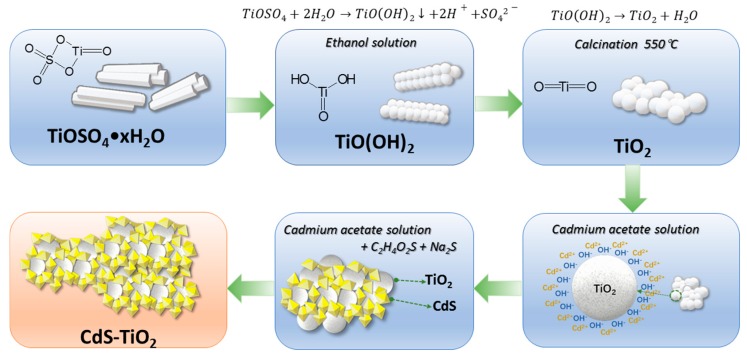
Schematic of preparation route of CdS-TiO_2_ heterostructure composite through a two-step process.

The principle of the morphology evolution is summarized in Figure 5. A dissolution-recrystallization process explains the reaction: Although the long rod-like precursor TiOSO_4_ dispersed in the ethanol solution, there was still a small amount of water, which was released from the crystal water (TiOSO_4_·*x*H_2_O). Subsequently, hydrolysis and alcoholysis were triggered under condition of high pressure and high temperature in the autoclave [42]. It is assumed that some of O-Ti-O bonds in TiOSO_4_ were broken during the solvothermal reaction and the TiO(OH)_2_ precipitated via hydrolysis reaction. Because limited hydroxide radical was provided from the crystal water, the product could not develop but became a nanoparticle in situ. As the reaction progressed, the three-dimensional framework of the raw material decomposed and the long rod precursor TiOSO_4_ disappeared, replaced by blocks composed of numerous nanoparticles. Anatase TiO_2_ was obtained after calcination at 550 °C for 3 h.

It is well known that hydroxyl radicals can be absorbed onto a TiO_2_ surface in aqueous solutions. Moreover, cadmium ion can be drawn to a TiO_2_ surface in the presence of hydroxyl. When S^2−^ was introduced into the solution, CdS precipitated out and attached to the TiO_2_ particles. In the meantime, a CdS-TiO_2_ heterostructure was generated, composed of two components of similar size.

### 3.2. Properties of CdS-TiO_2_ Composite

#### 3.2.1. Light Absorption Ability of the CdS-TiO_2_ Composite

**Figure 6:**
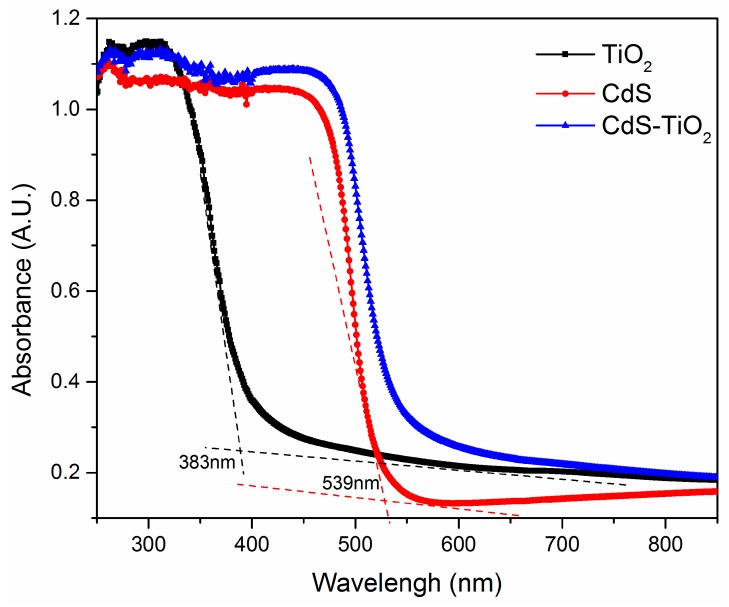
UV-vis spectra of the samples.

The diffuse reflection UV-vis absorption spectra of CdS-TiO_2_ heterostructure composite is shown in Figure 6, together with those of pure TiO_2_ and CdS as comparison. It is evident that pure TiO_2_ nanoparticles could only absorb up to 410 nm, which mostly belonged to the UV region because of its wide energy band gap (3.2 eV) [43] and was unlikely to respond to visible light. In comparison, the absorption features of CdS nanoparticles could reach 530 nm arising from the band absorption [38]. After sensitization with CdS, the absorption edge of CdS-TiO_2_ composite processed an obvious red shift that broadened to about 550 nm, which showed strong absorption capability in the visible light region. The existing difference in absorption edge wavelength for pure TiO_2_ and CdS-TiO_2_ clearly indicates that the light absorption process of TiO_2_ was altered and that the photo-response of the CdS-TiO_2_ as greatly improved through sensitization with the CdS nanoparticles [31,44]. The band gap energy of pure CdS and pure TiO_2_ was estimated by the following formula [45,46,47].

$$E_{g}=\frac{1240}{\lambda_{\mathrm{onset}}}$$

where *E*_g_ is the band gap energy and *λ*_onset_ is the absorption onset determined by linear extrapolation from the inflection point of the curve to the baseline [41,48,49]. The band gap energy results are also shown in Table 1.

#### 3.2.2. Photocatalytic Properties of CdS-TiO_2_ Composite

**Figure 7:**
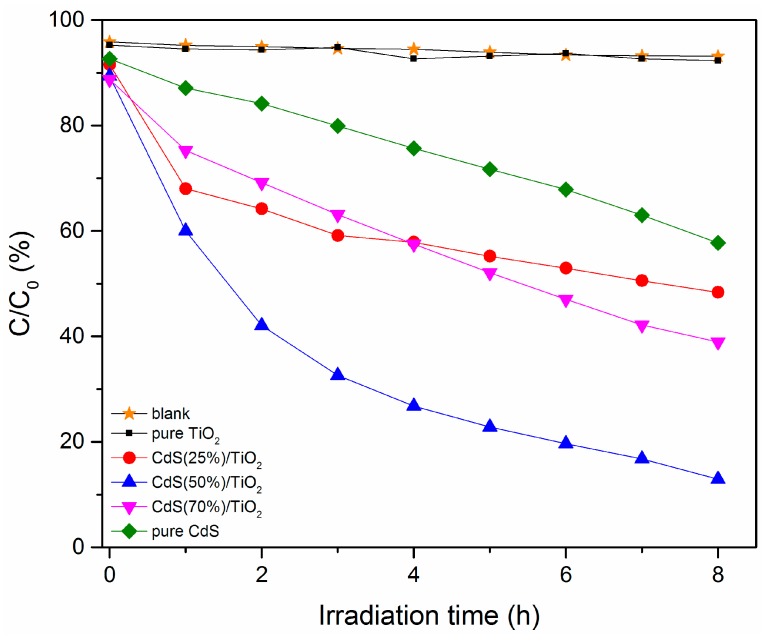
Degradation of tetracycline hydrochloride solution in the presence of different mass ration of CdS-TiO_2_ samples.

The photocatalytic activities of the CdS-TiO_2_ heterostructure as well as the sole TiO_2_ and CdS nanoparticles were evaluated through degradation of 50 mg/L tetracycline hydrochloride (TH) solution under visible light irradiation. Although the degradation effect of TH was affected by many conditions, such as pH, temperature, type of water matrix (ultrapure water, surface water, or groundwater), and so on, in this study, we only examined the TH degradation in ultrapure water. Figure 7 shows the concentration evolution of targeted raw material by the irradiation of visible light in the presence of different mass ratios of CdS-TiO_2_ samples (CdS: 0~100 wt %). Because TH is also sensitive to light, for comparison, we performed a blank experiment without any photocatalyst. As expected, the TH almost could not be degraded under the visible light. Additionally, the photocatalytic activity of pure TiO_2_ nanoparticles was also very low (7.68%). The degradation efficiency of TH-self and pure TiO_2_ was almost negligible. Obviously, the CdS-TiO_2_ composite, which had a 50% of CdS, showed the highest activity and the degradation of TH reached 87.06%. This phenomenon was explained by the fact that a higher percentage of CdS could absorb more visible light and yield an efficient transfer of excited electrons from the CdS nanoparticles to the conduction band of TiO_2_ nanoparticles [27]. However, when the mass percentage of CdS decreased to 25%, the degradation also decreased to about 51.64%. This was a result of fewer electrons able to be generated by CdS under visible light irradiation. However, the higher mass ratio of CdS (75%) also could not increase photocatalytic activity (61.04%). This may have been a result of a smaller TiO_2_ proportion, which both diminished the chance of photogenerated electrons moving from CdS to TiO_2_ and increased the charge recombination. This explanation also can be applied to the pure CdS nanoparticles in which the degradation was lower (42.26%).

**Figure 8:**
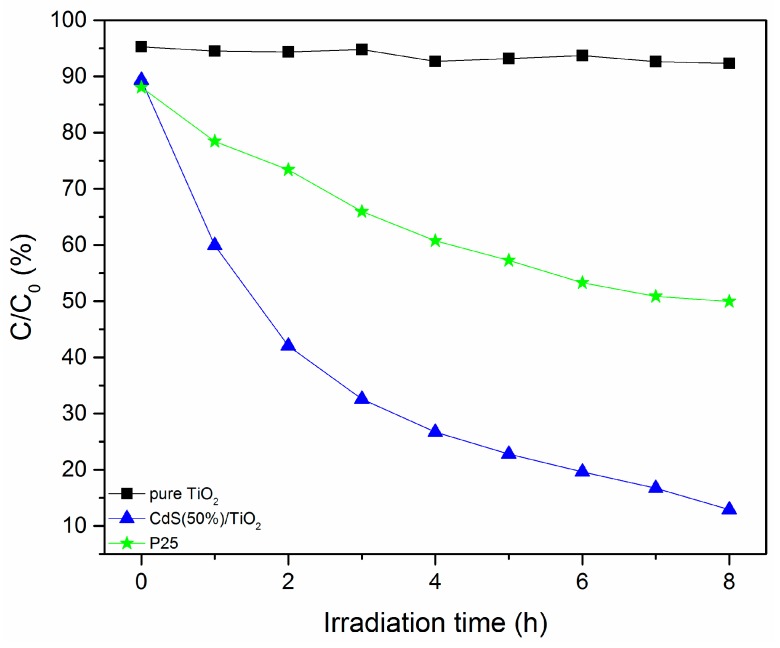
Degradation of tetracycline hydrochloride solution in the presence of CdS-TiO_2_ composites and P25 TiO_2_.

For further comparison as a reference material, the photocatalytic activity of the commercial TiO_2_ (Degussa P25) (Merck, Darmstadt, Germany) was also examined. As shown in Figure 8, the degradation efficiency could reach to 50% for P25 TiO_2_ after 8 h of photoreaction, which showed higher photocatalytic activity than the pure TiO_2_ synthesized in this study. Although both TiO_2_ are similar in morphology and structure, the commercial P25 TiO_2_ particles were composed of two kinds of crystal shape: anatase and rutile TiO_2_, which is easy to utilize the visible light when compared with the single-phase TiO_2_ [29]. However, the composite catalyst displayed much greater photocatalytic activity after the CdS was introduced. The great improvement of photocatalytic activity of the CdS-TiO_2_ heterostructure composite can be understood as follow: because the properly aligned conduction bands (CB) existed in the CdS-TiO_2_ composite, CdS nanoparticles could harness the visible light and effectively transfer photogenerated electrons to the CB of TiO_2_ [20]. In so doing, TH could be degraded much more easily than the P25 TiO_2_ degradation method.

#### 3.2.3. Mechanism of the Photodegradation by CdS-TiO_2_ Composite

**Figure 9:**
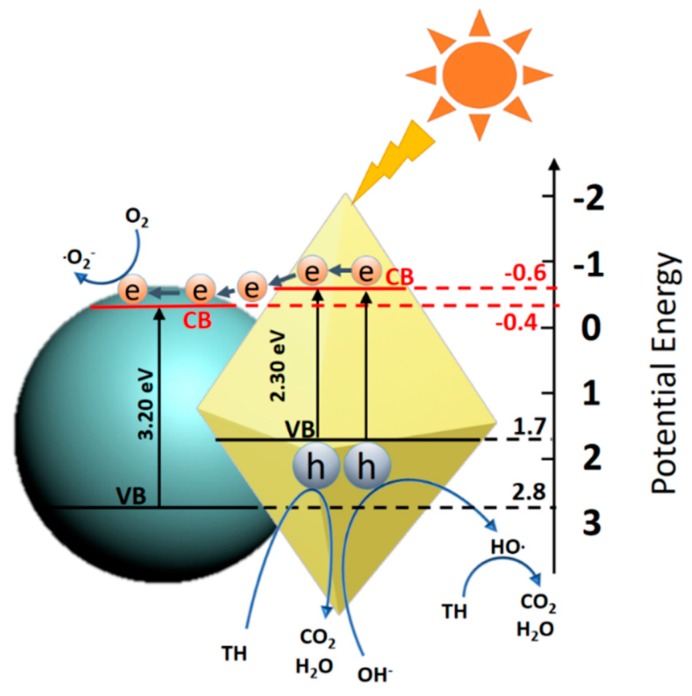
Schematic of the direct Z-scheme charge-carrier transfer process in the CdS-TiO_2_ heterostructure composite.

The photodegradation mechanism of organic molecules in CdS-TiO_2_ heterostructure composite system has been discussed in previous studies [26,27,29,50]. As shown in Figure 9, the CdS-TiO_2_ heterostructure composite featured the direct Z-scheme charge carrier transfer process [51]. Because of the lower band gap (about 2.3 eV), the carrier of CdS could be excited from the valence band (VB) to the conduction band (CB) by the visible light irradiation. When combined with the more positive CB of TiO_2_, the photogenerated electrons could be transferred from CB of CdS to that of TiO_2_, leaving high oxidation capability vacancies in the VB of CdS which could directly degrade TH. Simultaneously, these vacancies could be trapped on the surface of the photocatalyst, promoting the splitting of adsorbed water molecules or OH- forming hydroxyl radicals (·OH). These radicals have been considered as a type of strong oxidizing agents in which TH can also be oxidized during the photocatalytic reaction [52]. Similar-sized CdS nanoparticles were located on the surface of TiO_2_ nanoparticles, enlarging the contact surface area. The photogenerated electrons in the CB of CdS could efficiently transfer to the CB of TiO_2_ at the interface, easily generating high energy holes and electrons, which allowed for good utilization of visible light [43].

## 4. Conclusions

In summary, a CdS-TiO_2_ heterostructure composite was produced through a simple hydrothermal method by using TiOSO_4_ as a titanium precursor. In this composite, the CdS nanoparticles were uniformly loaded onto the surface of an anatase TiO_2_ nanoparticles. Both kinds of particles were similar in size. In comparison with the as-prepared pure TiO_2_ and Degussa P25, the CdS-TiO_2_ composite exhibited higher photocatalytic activity for tetracycline hydrochloride degradation, which reached 87.06% under visible light irradiation. The enhanced activity for the CdS-TiO_2_ composite was attributed to the more effective transfer of the photogenerated electrons due to the larger contact area between the two semiconductors.

## Figures and Tables

**Table 1:** Crystal size and band gap energy of the samples.

Sample	Crystal Size of Nanoparticles Calculated by the Peak Width (nm)	Band Gap Energy (eV)
CdS	19	2.30
TiO_2_	11	3.24
CdS in CdS-TiO_2_	22	-
TiO_2_ in CdS-TiO_2_	12	-
